# Perceptions of service providers, service recipients and female community health volunteers on a rural obstetric ultrasound program in rural Nepal: a qualitative study

**DOI:** 10.1186/s12884-023-05876-z

**Published:** 2023-08-10

**Authors:** Christine Kim, Kusum Wagle, Bhagawati Shrestha, Surya Bhatta, Sajana Maharjan, Liladhar Dhakal, Rajiv Rizal, Sibylle Kristensen

**Affiliations:** 1 One Heart Worldwide, San Diego, USA; 2 One Heart Worldwide, Lalitpur, Nepal; 3 AMDA-Nepal, AMDA Hospital, Damak, Jhapa, Nepal

**Keywords:** Rural ultrasound, Obstetric complications, Portable ultrasound, Early screenings, Obstetric ultrasound training, Abnormal scans

## Abstract

**Background:**

In rural Nepal, where women face financial and geographic barriers in accessing ultrasound scans, the government initiated a Rural Obstetric Ultrasound Program (ROUSG) to train skilled birth attendants (SBAs) in rural birthing centers and expand access to routine ultrasound scans for local pregnant women. This study explores the perceived benefits and limitations of the training and implementation of this program.

**Methods:**

A qualitative study was conducted in 15 primary care facilities in Bhojpur and Dhading, two rural districts of Nepal. The research team conducted in-depth interviews with 15 trained SBAs and focus group discussions with 48 service recipients and 30 FCHVs to gain insight into their perceptions. All interviews and focus group discussions were recorded, reviewed, and manually coded into MS Excel.

**Results:**

Overall, our findings indicated that the ROUSG program was very well received among all our study participants, though critical gaps were identified, mostly during the training of the SBAs. These included insufficient guidance or practice opportunities during training and the challenges of implementing the mobile obstetric ultrasound service. Most importantly, though, our results suggest that the implementation of the ROUSG program increased access to prenatal care, earlier identification and referrals for abnormal scans, as well as reduced pregnancy-related stress. There was also a notable anecdotal increase in antenatal care utilization and institutional deliveries, as well as high satisfaction in both service providers and recipients.

**Conclusion:**

Our findings highlighted that while the training component could use some strengthening with increased opportunities for supervised practice sessions and periodic refresher training after the initial 21-days, the program itself had the potential to fill crucial gaps in maternal and newborn care in rural Nepal, by expanding access not only to ROUSG services but also to other MNH services such as ANC and institutional deliveries. Our findings also support the use of ultrasound in areas with limited resources as a solution to identify potential complications at earlier stages of pregnancy and improve timely referrals, indicating the potential for reducing maternal and neonatal morbidities. This initial study supports further research into the role ROUSG can play in expanding critical MNH services in underserved areas and improving broader health outcomes through earlier identification of potential obstetric complications.

## Introduction

Widely used to determine gestational age, obstetric ultrasound scans have also been used to identify potential complications and high-risk pregnancies at an early stage, enabling prompt referral to appropriate higher levels of care [1]. Early detection of obstetric complications such as placenta previa for example, is of particular importance in improving both maternal and neonatal outcomes [2]. Studies in several low and middle-income countries (LMICs) have noted improvements in both the quality of care for obstetric conditions [3] and patient management [4] through the introduction of obstetric scanning. For high-risk pregnancies in LMICs, early screenings with obstetric scans have allowed healthcare providers to deliver more targeted follow-up and facilitate early referral to higher levels of care when needed [5]. In Nepal, obstetric scans provided by a trained radiologist are a paid service only available at tertiary-level urban hospitals and a few secondary-level district hospitals. As a result, low-income women living in remote rural areas with few available transportation options have very limited access to obstetric scans, resulting in potentially dangerous delays in identifying obstetric complications [6].

In 2011, in an effort to expand obstetric care to rural women under the Safe Motherhood Program, the Government of Nepal initiated a Rural Obstetric Ultrasound (ROUSG) program in two districts [7]. This program provides twenty-one days of obstetric scan training and a portable ultrasound to skilled birth attendants (SBAs), providing maternal and newborn health (MNH) services in rural areas. The ROUSG training is organized by the National Health Training Centre (NHTC) at the ROUSG training site and is provided by two radiologists, one Doctor of Medicine in General Practice (MDGP) doctor, and three ROUSG-trained nursing staff. The preferred trainer is a radiologist, followed by an MDGP with a radiology elective. ROUSG training sites are accredited by meeting five criteria: four facilitators to support training, a 1:2 facilitator-to-learner ratio with no more than 6 learners in a cohort, accreditation by the NHTC, an adequate caseload of USG services and obstetric cases per day, and sufficient infrastructure to support varied teaching methods. In Nepal, SBAs are primarily auxiliary nurse midwives (ANMs), or sometimes staff nurses, who have undergone an additional 2-months long in-service midwifery training [8]. Highly comparable to standard ultrasound machines, handheld and portable ultrasound machines have been found to be useful for rapid triage of obstetric emergencies [9], especially in areas with unreliable power sources and financial limitations [10]. The objective of the ROUSG training is to provide a basic level of obstetric ultrasound training to nurses at rural birthing centers to detect pregnancy-related complications for timely management and appropriate referral. The objective of the ROUSG program is to provide obstetric ultrasound scans to rural women by training nurses who are working as skilled birth attendants in rural public health institutions. These objectives are described in the 2022 Reference Manual for ROUSG Training for Nurses. Required core competencies of the program include operation/maintenance of portable ultrasound units, basic obstetric scanning techniques, identification of intrauterine gestation and fetal presentation /lie, the establishment of fetal viability, conducting fetal biometry to calculate gestational age and expected fetal weight, identifying normal and abnormal placenta, quantifying amniotic fluid and identifying abnormalities, maintaining records and reporting to appropriate facilities, and making appropriate and timely referrals for obstetric complications. Ideally, there should be at least one ROUSG-trained SBA for each palika (local municipality) where there is limited access to a trained radiologist. In rural areas of Nepal, most palikas operate 3 to 10 primary care facilities such as health posts and primary healthcare centers. The ROUSG-trained SBAs provide obstetric scans in the facility where they are based, but also expand service delivery by traveling to their palika’s other primary care facilities that lack regular ROUSG-trained providers. These services provided at the other facilities are termed “mobile ROUSG services” and are generally conducted at 2-3 facilities each month.

As part of a broader investment to improve the quality of MNH services and expand obstetric ultrasound infrastructure, a number of international organizations support the implementation of the ROUSG program in Nepal, including One Heart Worldwide (OHW), an international non-profit organization working in maternal and newborn health. Between 2015 and 2019, One Heart Worldwide alone sponsored 58 SBAs in rural birthing centers for training and provided 61 portable ultrasounds to improve antenatal care across 12 districts, namely Baglung, Bhojpur, Dhading, Illam, Khotang, Nuwakot, Okhaldhunga, Panchthar, Sankhuwasabha, Sindhupalchowk, Taplejung, and Terathum. Despite the growing demand for ROUSG service delivery and the increasing availability of the program throughout Nepal, there remains limited research on this specialized training for SBAs in rural birthing centers [11]. Subsequently, we conducted qualitative assessments among trained SBAs, service recipients, and local female community health volunteers (FCHVs) to provide us with a contextualized understanding of the perceived benefits and limitations of the ROUSG program in two rural districts of Nepal. FCHVS are local volunteers present in every community of Nepal. They promote healthy behaviors and access to local healthcare services by providing health education, conducting Health mothers group meetings, and distributing basic health commodities such as vitamins and iron. This study ultimately aims to support our assessment of the OHW-sponsored ROUSG program in other OHW program districts, gain greater insight into its successes and limitations from the perspective of the ROUSG program participants, and provide recommendations to improve the program within the Nepali context.

## Methods

### Study design

**Fig. 1:**
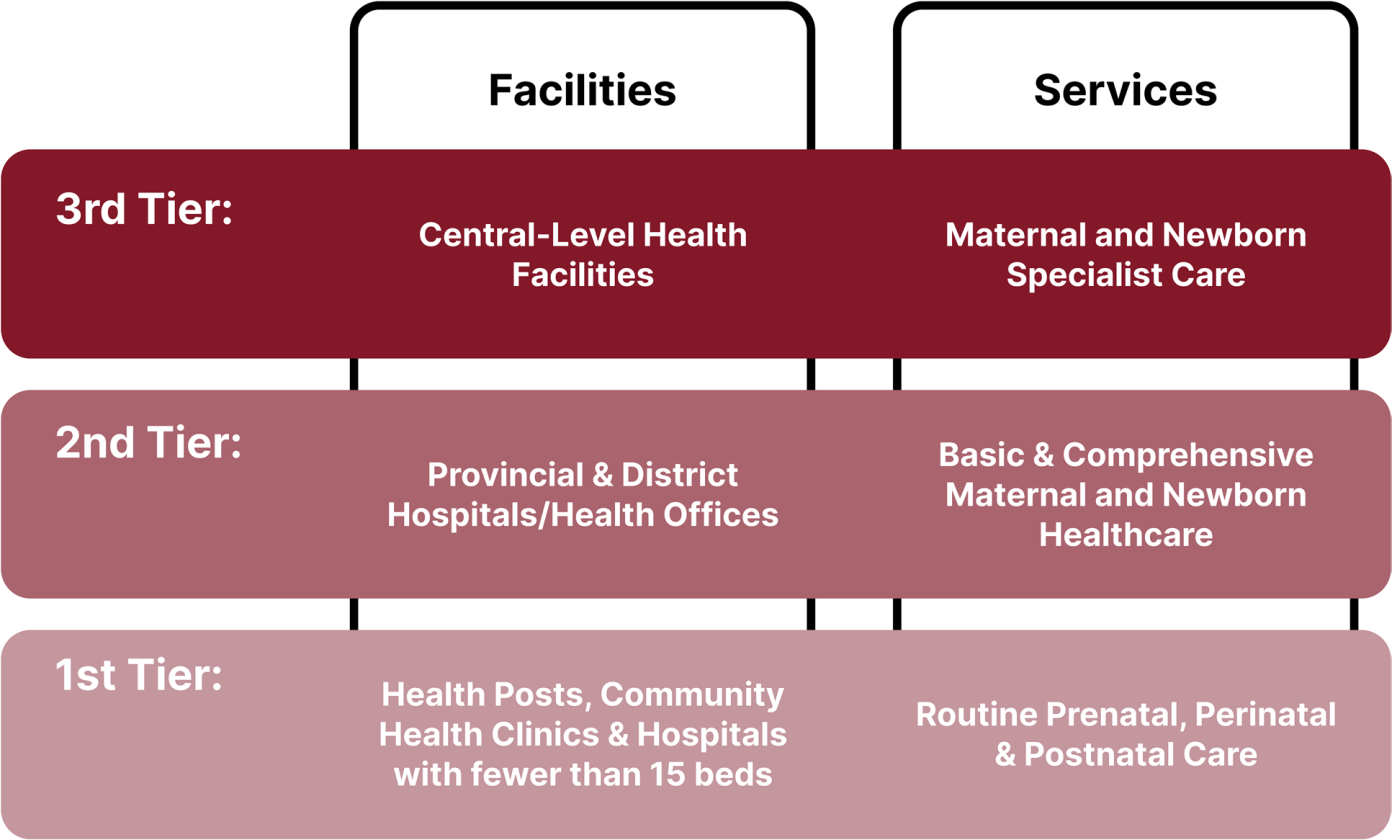
Division of Nepal’s Maternal and Newborn Care. The healthcare system in Nepal is divided into three tiers of facilities and their respective services for maternal and newborn health. The scope of the ROUSG training and program is within the first tier of care

A qualitative study was conducted in 15 primary care facilities (1st tier, see Fig. 1) in two districts of Nepal.

### Study setting

Among the 12 districts where OHW implemented the ROUSG program at the time of the study, the research team selected the two districts with the greatest number of available ROUSG-trained SBAs still using their portable ultrasound machine, specifically, Bhojpur and Dhading. In the Bhojpur and Dhading districts, OHW-sponsored ROUSG is the only organization-supported ROUSG program.

### Sampling

A non-probability convenience sampling technique was used for this study. Convenience sampling was used to select primary care facilities. Health facilities that had at least one ROUSG-trained SBA and were providing ROUSG services at the time of data collection were selected. For FGD with service recipients, we had purposely selected sites with high and low delivery. Thus, four FGDs were conducted with FCHVs and four FGDs with service recipients.

#### In-depth interviews with trained SBA/service providers

All 15 ROUSG-trained SBAs currently providing obstetric ultrasound services (10 in Dhading and 5 in Bhojpur) were included. For this study, ROUSG-trained SBAs are defined as ANMs or staff nurses employed by the Government of Nepal who have completed both the 2 months of in-service SBA training as well as the 21 days of ROUSG training.

#### Focus group discussions (FGDs) with service recipients

For each district, we purposely selected two primary care facilities among the 15 primary care facilities with a ROUSG-trained SBA to include one with a high flow number of ROUSG service recipients and another with a low flow number of ROUSG service recipients. Facilities with different flows were specifically chosen to ensure representation from facilities with varied rates of service utilization. A total of 48 service recipients, 12 per facility, were recruited for participation in the study. Inclusion criteria required the participants to be pregnant and have received an obstetric scan from the facility. A total of four FGDs were conducted.

#### FGDs with Female Community Health Volunteers (FCHVs)

In each district, we randomly selected 2 primary care facilities among the 15 primary care facilities with a ROUSG-trained SBA. The research team asked the ROUSG-trained SBA to reach out to all the FCHVs currently active in their catchment area. A total of 30 FCHVS were recruited from the catchment areas of four facilities, 17 from Bhojpur and 13 from Dhading. Inclusion criteria required the participants to be active FCHVs working in the catchment area of one of the four primary care facilities selected for the FCHV FGDs. A total of four FGDs were conducted.

### Data collection

Four research assistants (RAs) were recruited and trained for this study. All the data collection was conducted in May 2019.

#### In-depth interviews

The research team developed an interview guide including questions related to the SBAs’ perception of ROUSG training, their self-rated confidence in recognizing potentially abnormal scans, their perceptions of ROUSG program provision post-training, as well as suggestions to improve the program. For the self-rated confidence assessment, the SBAs were asked about their perceived ability to recognize an abnormal scan, as this is the purpose of the ROUSG training. As per the ROUSG Facilitators guide, abnormal scans include abortion, ectopic pregnancy, molar pregnancy, intrauterine fetal death (IUFD), low-lying placenta, an abnormal amount of amniotic fluid, and breech or transverse lie. ROUSG trainees are not expected to provide an actual diagnosis, as these are only provided by radiologists. To this effect, the SBAs were asked to rate their confidence from 1-4, where 1 represented “no confidence,” 2 as “low confidence,” 3 as “intermediate,” and 4 as “high confidence.” The RAs interviewed each of the ROUSG-trained SBAs during their visits to each of the 15 primary care facilities included in the study. All the interviews were recorded with the permission of the study participants to avoid missing any important information.

#### FGDs with service recipients and FCHVs

The research team developed separate FGD guides to facilitate discussions with the service recipients and the FCHVs. The FGD guide for service recipients included questions on their overall perceptions and experiences of the ROUSG services provided. The FGD for FCHVs included questions on their perceptions of the ROUSG services provided in their area and on service recipients’ reactions to ROUSG services. Four focus groups were conducted with the service recipients and four with FCHVs, one at each facility selected for this purpose. Each FGD was conducted by two RAs (one facilitating the discussion and the other taking notes) and recorded with the permission of the participants. The audio recordings were subsequently used to supplement the notes taken during the discussion while developing the FGD transcripts.

### Data analysis

The research team reviewed and translated the transcripts of the in-depth interviews and the FGDs from Nepali into English. They manually coded the translated transcripts using a recording format prepared in MS Excel with themes and codes derived from our study objectives as well as from the initial review of the transcripts. They concluded the process by conducting a thematic analysis of the data.

## Results

### Characteristics of study participants

#### Trained SBA/service providers

All 15 SBAs were stationed in primary care facilities. Twelve were trained as ANMs (an 18-month degree), and 3 were trained as staff nurses (a 3-year degree). Participants’ professional experience ranged from 2.5 to 23 years with all participants having worked at their current facility for at least 2 years. Fourteen provide daily ROUSG services at their primary care facility, while one runs the district’s maternal and child health (MCH) clinic, which does not offer ROUSG services onsite. All participants provide mobile ROUSG services, ranging from 2-3 times per month to a quarterly basis, to ensure that all women in their catchment areas have access to obstetric scans.

#### Service recipients

The 48 service recipients were pregnant women who had all received obstetric scans from the facility on the day of the FGD and none resulting in an abnormal scan. There were no specific pre-set selection criteria for when the ultrasound was conducted. Eight of the participants were in their first trimester, 24 were in their second trimester, and 16 were in the third trimester of pregnancy.

#### FCHVs

Of the 30, 5 FCHVs could read and write but had no formal education, 4 had a primary-level education, and 21 had some form of secondary education.

### Perceptions of the ROUSG training program

#### Positive perceptions

Almost all the SBAs (12) expressed satisfaction with the ROUSG training program and with the skills and abilities they gained in the process.

*“The training was excellent on the part of the skill development and case finding.”*

- SBA1-ANM

*“The training was very good and extra for me. Earlier, I had only heard about the obstetric scanning service but now being trained to perform the scan by myself makes me so happy.”*

- SBA13-ANM

#### Negative perceptions

Two-thirds of the SBAs (10) felt the training was too short and did not provide sufficient opportunities for hands-on practice. Participants also highlighted the lack of abnormal cases available to practice on.

*“There was a lot of confusion and time for practice was inadequate. We mostly saw normal cases.”*

- SBA7-ANM

*“The training was useful but the duration of training was short.”*

- SBA2-ANM

Half of the SBAs (7) specifically mentioned a lack of supervision during practice sessions due to the radiologists often being unavailable for the practice sessions. The SBAs emphasized the need to apply the learned conceptual material to physical practice under the continued guidance of the trainers and felt that the training lectures were disconnected from the practice sessions.

*“The doctor should manage more time for the training. We had to take our own patients and perform obstetric scans so it was difficult.”*

- SBA5-ANM

*“The practical and theory sessions were not conducted side by side. And the doctor was busy. so, we practiced by ourselves. He didn’t have time to teach everything.”*

- SBA4-ANM

The language used by the trainees led us to believe that a majority of the trainees may have had some misaligned expectations about the training, being under the impression that they should have been able to identify specific complications post-training versus being able to identify abnormal scans as per the training goals.

*“I have confusion in identifying ectopic pregnancy and length of the cervix.”*

- SBA13-ANM

*“Difficult to measure parameters when twins are full term and also difficult to detect placenta previa.”*

- SBA11-ANM

### SBA’s self-rated confidence in identifying abnormal scans

All ROUSG-trained SBAs were asked to self-rate their perceived confidence level in their ability to identify potentially abnormal scans. Among the 15 trained SBAs in the two districts, only three reported feeling highly confident in their ability to identify abnormal scans. The majority (11) shared that they were somewhat confident, while one shared having low confidence. They cited a lack of sufficient hands-on practical experience during training as the reason for their uncertainty.

*“I feel a bit scared to identify the abnormal scans.”*

- SBA9-SN

*“Theoretically all the content was covered but practical sessions were not adequate.”*

- SBA7-ANM

However, all the SBAs stated that when faced with any confusion or uncertainty during their scans, they were able to get support either by reaching out to a radiologist (including their trainers and/or the local hospital radiology team) or via a professional social media group for ROUSG-trained SBAs organized by OHW, where an OHW staff member was then able to link them to the support they needed.

*“I will reach out to the district hospital and ask for help from the district hospital staff (radiologists), the Facebook group, or directly contact the radiologist who trained us. I also refer to the manual for any confusion.”*

- SBA13-ANM

### Suggestions to improve the ROUSG training component for SBAs

A key piece of the feedback mentioned by two-thirds (9) of the SBAs was the need to improve the practice sessions. All of them requested better supervision from trainers during practical scanning practice sessions and refresher training sessions.

*“Trainers should give time to teach, guide, and supervise during the practice sessions as well.”*

- SBA1-ANM

*“Refresher training of one week by a radiologist should be provided. Similarly, onsite coaching would also be great.”*

- SBA11-ANM

Two of the SBAs suggested that each training participant should have their own ultrasound machines during the training session as opposed to the current practice of sharing devices to increase individual practice time.

*“It would be better if the trainer taught us from our machine and we had the chance to use our own machine for the training because we had to share the machine, so it was difficult.”*

- SBA5-ANM

Another two SBAs suggested having access to a training manual in Nepali (as opposed to the current English-language version) which would improve comprehension and minimize confusion.

*“Language of the training manual must be made more clear and it would be better if the manual was written in the Nepali language.”*

- SBA1-ANM

Four SBAs suggested that the training should be expanded to more SBAs so that each facility would have sufficient trained staff to improve the consistency of ROUSG services during staff leave time and have dedicated staffing to continue providing services from the facilities.

*“There should be 2 trained staff in one facility, 1 would run the mobile clinic and one would provide service inside the health facility.”*

- SBA11-ANM

*“It would have been better if there were two trained nurses in one facility so that in the absence of one of them the other can provide the service.”*

- SBA6-ANM

### Perceptions of the ROUSG services delivery

#### Increased access to obstetric scans for rural women

All the SBAs perceived the ROUSG services to be beneficial to pregnant women from lower socio-economic rural communities who lived too far away from the larger care facilities with a radiologist, considering that most of these women could not afford to travel to these larger care facilities.

*“It is very helpful, especially to women from the most remote and underprivileged communities in the districts. They have been able to get the services at their doorstep which they would not get otherwise.”*

* -* SBA2-ANM

*“Earlier, we had to send the clients to private hospitals for simple ultrasonography scanning which would cost 1,000 Nepali rupees but now that cost has been saved.”*

- SBA5-ANM

Eleven SBAs and seven service recipients stated that the mobile ROUSG services were particularly convenient and improved accessibility for pregnant women who did not live near primary care facilities with a trained SBA.

*“Pregnant mothers from far rural areas have difficulties getting obstetric scans. But, through this mobile service, many of these women benefited.”*

- SBA15-ANM

Service recipients shared that the availability of obstetric scans at nearby facilities increased their ability to access services and reduced the burden of extensive travel previously require to reach the district headquarters for scanning. Because the ROUSG services are provided free of cost to all women, participants felt it reduced the financial impact, not only related to expensive hospital scans but also to other travel and accommodation-related costs. A woman said,

*“Everything is available here. Earlier, we had to go to tertiary facilities for scans which are far. And now, we only need to go there if it is serious. As a result, the cost of going to other places has also been saved.”*

- PW3

Another service recipient said,

*“I went to the District Hospital for an obstetric scan and the total cost was 1600-1700 Nepali rupees. I had to travel for 1 day to go to the district hospital and 1 day to return. Through this ROUSG program, my time and money were saved. So, this ROUSG service is very advantageous to pregnant mothers like us.”*

- PW7

Similar perceptions were shared by all the FCHVS:

*“All pregnant women are happy with the obstetric scanning services. Some pregnant mothers say before this service started they had to go to a health facility far away, this way money, as well as time, is saved so they are happy.”*

- FCHV3

#### Improved ability for earlier identification and referral of high-risk cases

All SBAs shared that the obstetric scans helped them better recognize high-risk cases earlier in the pregnancy and refer these abnormal scans in a timely manner. Being proactive about referring abnormal scans allows potential complications to be detected early enough to ensure both service recipients and babies can be saved, something that was much more difficult to accomplish prior to the implementation of the ROUSG program.

*“It has become easier to identify high-risk cases due to the use of ultrasound scans. Earlier it was difficult to identify the fetal presentation, we could only do so by palpating because we had no ultrasound. We could only identify it at the last hour. The ROUSG program has helped in the early identification of high-risk cases and their early management.”*

- SBA6-ANM

#### Increased access to antenatal care (ANC) and institutional deliveries

Two-thirds of the SBAs (10) reported an increase in the number of women accessing ANC following the implementation of the ROUSG program. They explained that the ROUSG services created an opportunity to provide ANC to the service recipients who received ROUSG services but might not have come in for ANC services alone and increased trust in the ANC services.

*“Obstetric scanning has brought a positive impact on service seeking. The number of pregnant mothers for ANC check-ups has increased because of the obstetric scans now offered in this health post.”*

- SBA14-ANM

*“There is increased trust in the ANC service because of the obstetric scans.”*

- SBA1-ANM

Half (7) of the SBAs also mentioned an increased number of institutional deliveries due to the newly introduced obstetric scans at primary care facilities.

*“Yes, ANC visits and institutional deliveries have increased. The number of institutional deliveries has increased from 25 to 80 with the ROUSG program.”*

- SBA10-SN

#### Increased satisfaction among SBAs

Five SBAs expressed their satisfaction that service recipients who were referred for further follow-up responded positively and expressed gratitude towards the providers.

*“They (referring to service recipients) say that I did well by referring them.”*

- SBA10-SN

#### Increased satisfaction and reduced pregnancy-related stress among service recipients

All the SBAs mentioned that the service recipients were satisfied with their services and shared their appreciation for receiving referrals to higher-level primary care facilities when potential abnormalities were detected.

*“The mothers have a positive response after referral. The mothers thank us for saving their lives.”*

- SBA15-ANM

Service recipients expressed their relief in being able to visibly confirm the health status of their unborn baby. One woman shared,

*“In village areas, pregnant women have to do a lot of work like carrying jars of water and so on. In such situations, we come for a scan to know the position of the baby and identify whether the condition of the baby is good or not.”*

- PW7

Although service recipients receiving scans for the first time were nervous and scared before their exams, they expressed their contentment as soon as they knew that their unborn baby was in a healthy condition. They also stated that they felt reassured and less stressed about their baby after the scans.

*“Previously, I have had a miscarriage so I was curious and scared about why this is happening. So, I came here for an obstetric scan. After the scan, that sister (pointing toward the SBA providing obstetric scans) said that everything is fine. So I felt relaxed and happy.”*

- PW12

Six of the FCHVs stated that they felt that the obstetric scans were helping service recipients to know whether their unborn baby was healthy and growing as expected or if there were unexpected factors.

*“Pregnant mother says we do not know these things so we go to the health facility and find our baby’s condition.”*

- FCHV3

One service recipient reported that her previous exam to assess the position of the baby had been a manual exam but she felt that her recent obstetric scan was a more reliable method to determine her baby’s position. Another service recipient shared her satisfaction in receiving and following the advice given by the SBA after completing her scan, saying,

*“In my case, the baby was in the upper part and the placenta was lower so the sister advised me not to carry heavy things, and not to travel in vehicles.”*

- PW12

All the service recipients shared that they would recommend the SBAs’ services to other pregnant women.

*“Yes, we do suggest other women in the village do the obstetric scanning service.”*

- PW4

All the FCHVs reported that most service recipients in their areas understood the value of the obstetric scans, reacted positively to the availability of ROUSG scans, and were thankful for the benefits it provided them and their unborn infants.

*“Yes, every woman understands the importance of the scans and they also understand that it is beneficial for them.”*

- FCHV3

*“They react positively. They thank us for informing them about the obstetric scans. They express their gratitude towards these services for early detection of high risks and minimizing complications.”*

- FCHV2

#### Challenges specific to the mobile ROUSG program

Over half of the SBAs (8) reported facing challenges while implementing mobile ROUSG services in their municipalities. The main difficulty was related to transportation issues to reach some of the more remote facilities where they planned to offer mobile scans.

*“Long distance to travel to the health post, stiff and dangerous roads, lack of transportation facilities to reach the health post are some of the challenges.”*

- SBA14-ANM

Despite having allocated budgets for travel, one-third of the SBAs (6) reported difficulties obtaining the required travel funds from their local municipalities to support the implementation of the mobile ROUSG. Approval and distribution of the funding appear to be inconsistent amongst the service providers.

*“Since the Municipality has not allocated the budget, I am using my own personal funds to run the mobile clinics.”*

- SBA9-SN

Other issues limiting the mobile camps included power availability and staffing constraints. Continuing services at health posts are the priority thus no mobile clinics are organized if the birthing center has only one SBA. ROUSG mobile clinics are only run if at least two SBAs are available.

*“Problems in charging the ultrasound machine during rainy seasons because of electricity problems.”*

- SBA1-ANM

*“There is a staffing problem because I am the only one here who provides the obstetric scanning service and in my absence, the service is halted.”*

- SBA6-ANM

#### Reservations towards the ROUSG program

Though an overwhelming majority of service recipients and FCHVs reported positive perceptions of the SBAs and the ROUSG services, some of the FCHVs noted cases where service recipients showed reservations, especially toward referrals recommendations for abnormal scans.

*“It is not that the pregnant women do not want to go as referred. But mostly it is due to the family pressure of the women that influences her from acting on referral.”*

- FCHV2

*“Some families are cooperative and understanding whereas some do not understand and are narrow-minded. Some even believe that women should not travel during pregnancy.”*

- FCHV7

#### Negative reactions to obstetric scanning policy

Despite sex identification being illegal in Nepal and SBAs strictly prohibited from using obstetric scans for that purpose, 4 FCHVs shared that some service recipients had some negative reactions or questioned the usefulness of the procedure/integrity of the providers.

*“They say that if the sex of the baby is not determined, why should we do obstetric scans and ANC check-ups.”*

- FCHV2

*“Even when we explain why, they say that the sisters (SBAs) are lying intentionally, not telling them about the sex of the baby.”*

- FCHV7

## Discussion

The ROUSG program is a practical task-shifting approach from qualified radiologists to SBAs, specifically designed with shorter training and fewer qualifications as defined by WHO [12]. Similar programs have also been implemented in other low and middle-income countries like Nepal with high rates of success and accuracy [10, 13, 14]. Overall, our findings indicated that the ROUSG program was well received among all our study participants, though some challenges were identified, mostly during the training of the SBAs.

### Perceptions of the ROUSG training program

While most of the SBA trainees in our study expressed satisfaction with the ROUSG training program and all were appreciative of their newly acquired skills, a majority of the SBAs also felt that the theoretical and practical sessions were disjointed, thereby limiting their ability to fully engage with the material or apply their knowledge. More concerningly, a majority of the SBA trainees raised the very concerning issue of inadequate supervision during practical sessions, impairing both the quality and efficacy of the practical training sessions and risking reinforcement of incorrect scanning practices. Though a significant majority of the SBAs in our study felt that the 21-day training program was not long enough for them to gain a strong foundation in obstetric scanning, this particular feeling seems to be predominantly associated with the limitations of the available frequency and types of hands-on practical sessions than the actual training length. Comparable studies suggest that offering longer training sessions do not significantly impact the accuracy of the trainees’ scans, with widespread accuracy and high-quality scans reported across programs of varying lengths [15, 16]. As such, the trainees are taught how to conduct a basic ultrasound scan, with most of their practice exercises conducted on normal pregnancies. Because the availability of abnormal cases during a training session cannot be guaranteed, the trainers must use pictures and videos to supplement illustrations of abnormal scans. The ROUSG training program is not intended to teach SBAs how to diagnose complications, but instead to recognize abnormal scans and initiate prompt referrals. Thus, according to findings in other studies, these visual materials should be sufficient to enable SBAs to recognize abnormal scans [17]. The reported frustration regarding the lack of available complications on which to practice may also stem from misaligned expectations held by some trainees who felt that they should be able to not only accurately recognize abnormal scans but also correctly identify all potential complications. Several trainees felt that their training was inadequate without achieving the level of service that is only to be expected of a qualified radiologist. As trainees are not responsible for providing scanning to the level of radiologists, their expectations were misaligned with the government’s own expectations for this type of training program [18]. 

The training limitations were clearly reflected in the self-rated confidence levels reported by the trained SBAs. Though most of them felt at least somewhat confident in recognizing a potentially abnormal scan, only three felt highly confident in these capabilities. When queried, all the SBAs identified the lack of practice opportunities for abnormal scans and inadequate supervision during practice sessions as their lack of confidence. During service delivery, they reported reliance on the radiologist on staff and professional social media groups to address uncertainty about a scan’s results.

Suggestions offered by the SBA trainees to improve the program were predominantly focused on improving the quality of the practical component of the initial training. It is essential that all practical sessions have reliable supervision and effective guidance throughout each session when applying practical skills to ensure the trainees are learning correct scanning techniques and can correctly conduct high-quality, accurate scans. Ensuring access to individual portable ultrasound machines for each trainee could further enhance the productivity and efficiency of the practical sessions by enabling each SBA to practice on her own device, which would amplify the opportunities to practice and enable the trainees to further familiarize themselves with the devices and increase their comfort using them.

Specifically regarding the training manual [19], many trainees requested the English curriculum to be translated into Nepali for easier understanding and comprehension. Clarifying the purpose of the training upfront and translating the training manual may also preempt misunderstandings and better manage trainees’ expectations of the training program. Other suggestions included adding refresher sessions post-training as trainees felt the need for recurrent practice time, a perspective supported by a study in Tanzania that linked increased hands-on practice during training with improvement in trainees’ skills retention and utilization [20]. Refresher training sessions would provide opportunities for trainees to practice scanning and gain more experience with abnormal scans under appropriate supervision and refresh the theoretical knowledge acquired during their initial training program. Due to differences in patient flow, there is variability in the scanning frequency and the type of complications addressed per SBA after training is complete. Providing refresher training sessions would not only improve support and ongoing education to build confidence and address uncertainty about abnormal scans but also encourage trainees to stay up to date with their skills and training knowledge regardless of their scanning frequency. The outcomes of implementing these suggestions would also address staffing concerns by producing more knowledgeable ROUSG-trained SBAs to ultimately improve the availability of scans and expand service regions to increase accessibility to more rural Nepali women.

### Perceptions of the ROUSG services delivery

All study participants felt that increasing access to free obstetric scans at local primary care facilities was very beneficial for pregnant women in rural areas who usually face major financial and transportation limitations to access paid obstetric scans in larger hospitals.

#### SBAs

All the SBAs enrolled in our study felt that the ROUSG program had improved their capacity to provide better obstetric care to rural pregnant women, as their new ability to identify abnormal scans allows potential complications to be detected early enough to ensure both mothers and babies can receive appropriate care and be spared complications and adverse events. In addition, two-thirds of the SBAs reported that the availability of obstetric scans had also increased the number of women attending ANC visits, serving as a motivator for women who might not have sought ANC previously. Half of the SBAs also reported an increase in institutional deliveries, suggesting a correlation between the obstetric scans and the increased ANC visits as seen in similar studies [21, 22]. The increased ANC attendance and institutional deliveries suggest added potential for improved health outcomes for both mothers and babies as a result of the ROUSG program. The mobile ROUSG program was perceived as particularly beneficial for marginalized women living in more remote areas. All the SBAs in our study reported positive experiences from their interactions with their patients when providing ultrasound scans. The ROUSG program was perceived as a positive professional achievement and all SBAs expressed pride and satisfaction in their newfound ability to provide critical specialized skills for their communities.

The SBAs did report several difficulties related to the mobile ROUSG program, including transportation, finances, unreliable power, and staffing shortage. Transportation was a significant challenge due to poor road conditions, difficulties securing reliable transportation to remote communities, and a lack of sufficient travel funds from local authorities. Despite verbal agreements for mobile service funding from the local government, many SBAs reported that funds were not provided in advance of their travels, requiring them to use personal funds to maintain the mobile clinics. Additionally, unreliable power sources for the ultrasound equipment and an insufficient number of ROUSG-trained SBAs to run both the mobile clinics and the in-facility scanning services threatened the consistency and continuity of services. Local governments wanting to implement the ROUSG program and expand access to care will need to invest, not only in the training of additional SBAs but in the infrastructure and systems essential to effective service delivery. Additionally, the program may need to explore alternative power solutions such as a solar backup or battery pack to power the ultrasound (USG) machine when the power is unavailable as it so often is during the winter and monsoon seasons.

#### Service recipients

All the service recipients in our study reported positive experiences utilizing ROUSG services. They all felt that obstetric scans were more reliable than the standard manual exams for pregnancy assessments. They also expressed their relief at being able to visibly confirm the health status of their babies and alleviate their personal concerns and pregnancy-related stress, findings consistent with similar studies in other LMICs [11]. Service recipients also reported increased satisfaction with both service providers and service delivery post-ROUSG service implementation, another finding consistent with other studies [11, 23]. The interpersonal relationships between the service recipients and the SBAs further reflected the service recipient’s satisfaction with receiving individualized scanning time and feedback. All service recipients shared that they would recommend obstetric scanning services to other pregnant women and they frequently cited the convenience of having greater physical access to the services as a strong motivator for receiving obstetric scans.

#### FCHVS

The FCHVs reported that most of the service recipients had a positive perception of the SBAs and the obstetric scans. The FCHVs noted that the mother’s positive perception largely aligned with the reported reduced pregnancy-related stress and improved access to obstetric scans regardless of financial or geographical limitations reported by SBAs. Both FCHVs and SBAs reported frequent expressions of appreciation by service recipients towards the obstetric scans and showed a positive response to referrals for abnormal scans. Interestingly, the FCHVs were the only group that reported a couple of factors that sometimes negatively influenced service recipients’ experiences. Though all study participants reported that most service recipients usually followed the SBAs’ referral advice, only the FCHVs reported that some of the service recipients faced opposition from their families when given a referral recommendation based on traditional beliefs that discourage medical intervention during pregnancy. The FCHVs also reported frustration from some service recipients when reminded that in Nepal, by law, obstetric scans could not be used for sex identification. As such, it is important for SBAs to be cognizant of local contexts and to include family members as much as possible when providing education and recommendations for the health of the mother and/or her unborn infant.

### Study limitations

Our results are not generalizable to other locations or populations since qualitative research is not based on random samples and statistical controls. This study is solely based on the perceptions of the participants which may not be supported due to a lack of evidence-based data. Additionally, it is noteworthy to mention that there was no representation of a potential complication or referral among our FGD with service recipients. Reflecting the use of convenience samples, participants were selected from the service recipients who had received an obstetric scan at the primary care facility that day, none of whom had any complications. The wording of the questions did not always ask for specific perceptions about individual components of the ROUSG training, potentially causing confusion or misleading participants in their answers. The quality of the ultrasound examinations conducted by the participants was not thoroughly assessed to address false positive and false negative results.

## Conclusions

This study evaluated ROUSG training and service provision solely based on the reported perceptions of service providers, service recipients, and community health volunteers. Overall, the ROUSG program was positively perceived among our study participants. This study does suggest that the ROUSG training for non-radiologist providers could be more effective by including more extensive hands-on practice in normal cases, better supervision from the NHTC certified ROUSG radiologist trainers, and post-training mentoring as a part of CME to maintain and gain new skills and improve confidence. The recommendation to implement these sessions for post-training after the initial 21-day training is emphasized due to the current lack of any post-training system to conduct clinical mentoring and onsite coaching. Despite the fact that this study only addresses the perceptions of the ROUSG training recipients and lacks quantifiable evidence, current literature does indicate that their perceptions are correct and that they would indeed benefit from additional hands-on and refresher training. Though there is no quantifiable evidence, our findings suggest that the ROUSG program has the potential to fill crucial gaps in maternal and newborn care in rural Nepal, by expanding access not only to ROUSG services but also to other MNH services such as ANC and institutional deliveries. Our findings also support the use of ultrasound in areas with limited resources as a solution to identify potential complications at earlier stages of pregnancy and improve timely referrals indicating the potential for reducing maternal and neonatal morbidities. This is particularly relevant within the context of resource-constrained countries like Nepal where ultrasound services are limited to secondary or tertiary-level facilities. Our findings also highlighted the need for increased support regarding the mobile ROUSG program, particularly in terms of travel costs, available infrastructure, and sufficient staffing to maintain both onsite and mobile ROUSG services. Further research is needed to assess knowledge and skill retention post-training by assessing the trainee’s ability to correctly recognize potentially abnormal scans and the impact of the ROUSG program on the management of complications and adverse obstetric outcomes.

## Data Availability

The datasets and tools used for this study are available from the corresponding author upon reasonable request.

## Abbreviations

ANC: Antenatal Care
ANM: Auxiliary Nurse Midwives
FCHV: Female Community Health Volunteer
FGD: Focus Group Discussion
LMIC: Low and Middle-Income Country
MCH: Maternal and Child Health
MDGP: Doctor of Medicine in General Practice
MNH: Maternal and Newborn Health
NHTC: National Health Training Centre
OHW: One Heart Worldwide
PW: Pregnant Woman
RA: Research Assistant
ROUSG: Rural Obstetric Ultrasound
SBA: Skilled Birth Attendant
SN: Staff Nurse
USG: Obstetric Ultrasound
